# Current insights into the relationship between the gut microbiome and Sjögren’s syndrome

**DOI:** 10.1186/s12934-020-01471-5

**Published:** 2020-11-13

**Authors:** Taco A. van der Meulen, Frans G. M. Kroese, Hendrika Bootsma, Fred K. L. Spijkervet, Arjan Vissink

**Affiliations:** 1 Department of Oral and Maxillofacial Surgery, University of Groningen, University Medical Center Groningen, Groningen, The Netherlands; 2 Department of Rheumatology & Clinical Immunology, University of Groningen, University Medical Center Groningen, Groningen, The Netherlands

Dear editor,

With interest we read the recent publication by Mendez et al. [1] entitled ‘Gut microbial dysbiosis in individuals with Sjögren’s syndrome’ in which the authors report that individuals with dry eye signs have gut microbiome alterations compared to healthy controls. They conclude that their study sets the foundation for gut microbiome modulation as a potential therapeutic target for patients with dry eye measures.

The aim of the study by Mendez et al. [1] was to evaluate the gut microbiome in patients with dry eye, with or without SS and to correlate microbiome profiles to clinical parameters, in general only related with dry eye. In their cohort of 21 healthy controls and 21 patients with dry eye signs, only 13 patients with dry eyes (62%) fulfilled the 2016 American College of Rheumatology criteria for SS [2]. Although Mendez et al. shortly discuss the heterogeneity of their patient population as a limitation of their study, it is unclear whether the group of SS patients was composed of only primary SS (pSS) patients or of a combination of primary and secondary SS (sSS) patients. Four out of 13 (31%) SS patients in their study were male patients, whereas in pSS the female:male ratio is 10:1 [3]. Furthermore, 23% of their SS patients (3 out of 13) had a comorbid autoimmune disease, which may indicate that these patients had sSS. Unfortunately, Mendez et al. [1] do not mention which autoimmune comorbid diseases these three SS patients suffered from. The possible mixture of pSS and sSS patients in their SS-group may have influenced the findings in the gut microbiome of their SS patients. The comorbid autoimmune diseases mentioned in the study of Mendez et al. [1] (i.e., rheumatoid arthritis, systemic lupus erythematosus, psoriatric arthritis and systemic sclerosis) are on their own also related to changes in the gut microbiome [4]. Thus, their SS patient group is heterogeneous and not representative for the average pSS or sSS population in the United States or Europe [5]. In addition to this heterogeneity, comparison with healthy controls is hampered by the notion that controls were all male and were younger than the patient groups. Sex and age both affect the composition of the intestinal microbiota [6].

The main difference Mendez et al. [1] observed in the gut microbial composition of SS dry eye (SDE) patients and non-SS dry eye (NDE) patients compared with healthy controls was a significant difference in the Unweighted-Unifrac Principal coordinate analysis (PCoA). However, the gut microbial composition of SDE and NDE patients did not differ significantly, suggesting that the dysbiosis in gut microbial composition in SS patients is not disease specific, but, e.g., related to dry eye signs. It would be of interest to apply essential comparative statistics to support and substantiate the dysbiosis seen by PCoA.

Several studies analyzed the gut microbiome in pSS patients [6–10], but for some reason Mendez et al. limited the comparison of their data only to the study by de Paiva et al. [7]. Mendez et al. stated that a similar decrease in relative abundance of Faecalibacterium and Bacteroides was found in both studies [1, 7]. However, de Paiva et al. [7] performed a pilot study comparing the gut microbiome from 10 pSS patients with data from 45 samples of healthy individuals who participated in the Human Microbiome Project. Direct comparison of microbiome samples between two different cohorts is highly at risk for false positive findings, because of methodological differences between cohorts, ranging from fecal sampling to DNA analysis.

Two other studies on gut microbiome in pSS reported a statistically significant higher relative abundance of phylum Bacteroidetes in the gut microbiome of pSS patients compared to controls [6, 9]. The observed tendency of a lower relative abundance of genus Bacteroides in pSS patients compared to controls in the studies by Mendez et al. [1] and de Paiva et al. [7] was not statistically significant, and markedly contrasted our own study showing significantly higher relative abundance of genus Bacteroides in pSS patients (n = 39) than in population controls (n = 965) [6]. Furthermore, we were able to identify three Bacteroides species (i.e., *B. vulgatus*, *B. uniformis* and *B. ovatus*) of which the relative abundance was significantly higher in pSS patients than in population controls [6]. Another Bacteroides species, *Bacteroides thetaiotaomicron* (*B. theta*), showed a tendency to be higher in pSS patients than in controls [6]. *B. theta* has been suggested as a potential gut pathobiont (i.e., a potential pathogenic micro-organism, which, under normal circumstances, is harmless) in patients with anti-Ro60 auto-antibodies [11]. Lysates of *B. theta* bind to serum from anti-Ro60-positive patients with systemic lupus erythematosus. Furthermore, B and T cell responses to the Ro60-protein occurred after monocolonization of mice with *B. theta*, subsequently leading to enhanced lupus-like disease in mice [11]. Because anti-Ro60 autoantibodies are observed in up to 70% of pSS patients, the findings of Greiling et al. [11] may suggest a potential role for *B. theta* in the pathogenesis for pSS also. However, there is no evidence for an association between the presence of anti-Ro60 auto-antibodies in serum and *B. theta* relative abundance in fecal samples of pSS patients or patients with systemic lupus erythematosus [6, 11]. Thus, there is currently more evidence supporting that a higher rather than a lower relative abundance of Bacteroides species is related to having pSS [1, 6, 9, 11].

Taken together, we conclude that we are far beyond drawing more definite conclusions about possible roles of particular bacterial species or groups of bacteria in the pathogenesis of SS (and dry eye disease). Future studies assessing the role of the human microbiome in pSS patients, should significantly increase in number of well-defined pSS patients [6]. Bacterial compositions on the ocular surface and in the oral cavity have been associated with pSS. Therefore, future studies should include not only gut, but also oral and ocular microbiome samples to obtain a complete picture of the microbiome – pSS connection [12].

## Patient and public involvement

Patients and/or the public were not involved in the design, conduct, reporting or dissemination plans of this research.
